# HIF-1-Independent Mechanisms Regulating Metabolic Adaptation in Hypoxic Cancer Cells

**DOI:** 10.3390/cells10092371

**Published:** 2021-09-09

**Authors:** Shen-Han Lee, Monika Golinska, John R. Griffiths

**Affiliations:** 1 Department of Otorhinolaryngology, Hospital Sultanah Bahiyah, KM6 Jalan Langgar, Alor Setar 05460, Kedah, Malaysia; 2 Cancer Research UK Cambridge Institute, University of Cambridge, Li Ka Shing Centre, Robinson Way, Cambridge CB2 0RE, UK; monika.golinska@cruk.cam.ac.uk (M.G.); john.griffiths@cruk.cam.ac.uk (J.R.G.); 3 Department of Physics, University of Cambridge, JJ Thomson Avenue, Cambridge CB3 0HE, UK

**Keywords:** hypoxia-inducible factor-1 (HIF-1), hypoxia, cancer metabolism, glycolysis, creatine metabolism, Myc, phosphatidylinositol 3-kinase (PI3K), AMP-activated protein kinase (AMPK), 2-hydroxyglutarate, glutamine metabolism, lipid metabolism

## Abstract

In solid tumours, cancer cells exist within hypoxic microenvironments, and their metabolic adaptation to this hypoxia is driven by HIF-1 transcription factor, which is overexpressed in a broad range of human cancers. HIF inhibitors are under pre-clinical investigation and clinical trials, but there is evidence that hypoxic cancer cells can adapt metabolically to HIF-1 inhibition, which would provide a potential route for drug resistance. Here, we review accumulating evidence of such adaptions in carbohydrate and creatine metabolism and other HIF-1-independent mechanisms that might allow cancers to survive hypoxia despite anti-HIF-1 therapy. These include pathways in glucose, glutamine, and lipid metabolism; epigenetic mechanisms; post-translational protein modifications; spatial reorganization of enzymes; signalling pathways such as Myc, PI3K-Akt, 2-hyxdroxyglutarate and AMP-activated protein kinase (AMPK); and activation of the HIF-2 pathway. All of these should be investigated in future work on hypoxia bypass mechanisms in anti-HIF-1 cancer therapy. In principle, agents targeted toward HIF-1β rather than HIF-1α might be advantageous, as both HIF-1 and HIF-2 require HIF-1β for activation. However, HIF-1β is also the aryl hydrocarbon nuclear transporter (ARNT), which has functions in many tissues, so off-target effects should be expected. In general, cancer therapy by HIF inhibition will need careful attention to potential resistance mechanisms.

## 1. Introduction

Oxygen is essential for the normal viability and function of eukaryotic cells due to its role in mitochondrial energy production and as a co-factor/substrate for many enzymes. Under conditions of low oxygen tension, normal cells undergo a metabolic switch from a predominance of mitochondrial metabolism towards increased glycolysis in order to maintain sufficient ATP levels [1]. Hypoxia then activates a signalling pathway which is predominantly governed by stabilisation of hypoxia-inducible factors, principally the transcription factor hypoxia-inducible factor 1 (HIF-1) [2,3], which activates a program regulating the expression of numerous genes involved in metabolic processes, including glycolysis [1], angiogenesis [4], pH regulation [5], and wound healing [6]. The significance of the discovery of how cells sense and adapt to oxygen availability via the HIF signalling pathways was recognised by the award of the 2019 Nobel Prize in Physiology or Medicine to William G. Kaelin Jr, Sir Peter J. Ratcliffe, and Gregg L. Semenza.

In solid tumours, uncontrolled proliferation of cancer cells and disorganised growth of blood vessels creates regions of low oxygen tension that limit the supply of nutrients and oxygen. The HIF-1 transcription factor is highly overexpressed in a broad range of human cancers and plays a critical role in driving tumour growth, invasion, and metastasis [7]. Due to the importance of HIF-1 in cancer, this pathway is a potential therapeutic target, with a number of HIF inhibitors currently under investigation in pre-clinical and clinical studies [8,9]. Several compounds are currently in phase II clinical trials, either as single agents or in combination with other anticancer agents, mainly for the treatment of advanced or refractory cancers [9]. Of note, a selective small molecule inhibitor targeting the HIF-2α transcription factor, Belzutifan (MK-6482), has been reported to reduce the size of tumours and inhibited tumour progression in almost 90% of von Hippel–Lindau (VHL) patients over a 3-year study period [10,11]. This led to a recent United States Food and Drug Administration (FDA) approval for Belzutifan in adult patients with VHL-associated clear cell renal cell carcinoma (RCC), central nervous system hemangioblastomas, and pancreatic neuroendocrine tumours [12].

The development of drug resistance remains a major challenge in anticancer therapies, and it is likely that this will be the case for HIF-1 inhibitors. Several preclinical models of HIF-1-deficient tumours initially undergo a period of slow growth before subsequently demonstrating accelerated growth, suggesting the development of resistance and escape mechanisms from the inhibition of HIF-1 [13,14,15,16]. Since HIF-1 is a well-recognized regulator of numerous metabolic pathways, particularly glycolysis, extensive metabolic adaptations will be required for cancer cells to survive under hypoxic conditions in its absence. Therefore, understanding the HIF-1-independent mechanisms that could regulate such metabolic adaptations in hypoxic cancer cells is crucial to circumventing the potential challenge of treatment resistance.

In the past two decades, advances in multi-omics technologies (genomics, transcriptomics, proteomics, and metabolomics) and experimental modelling of cancer metabolism have yielded new insights into metabolic adaptations of HIF-1-deficient cancer cells in hypoxia. In this review, we will examine the accumulating evidence suggesting that HIF-deficient cancer cells can activate alternative metabolic and signalling pathways to metabolically adapt to hypoxia. We will also review some of the other HIF-1-independent mechanisms that should be investigated in HIF-deficient cancer cells, with a focus on adaptations in glucose, glutamine, and lipid metabolism.

## 2. Glucose Metabolism & Hypoxia

To begin, we will briefly introduce the basic steps of glucose metabolism and examine how it changes under hypoxia. Glycolysis converts a single molecule of glucose into two molecules of pyruvate, with the net phosphorylation of 2 molecules of adenosine diphosphate (ADP) to adenosine triphosphate (ATP) and reduction of 2 molecules of nicotinamide adenosine dinucleotide (NAD) to NADH (reduced NAD) [1]. The fate of pyruvate is variable: it can be converted to lactate or carbon dioxide, depending on the availability of oxygen. In the presence of sufficient levels of oxygen, pyruvate is further oxidised by the tricarboxylic acid cycle (TCA) in the mitochondria, generating electron carriers to the electron transport chain which generate a further 36 molecules of ATP per molecule of glucose entering the glycolytic pathway [1]. In hypoxia, pyruvate remains within the cytoplasm and is converted to lactate in a reaction catalysed by lactate dehydrogenase [1]. This allows for the regeneration of NAD^+^ from NADH to maintain glycolysis during hypoxia [1]. High rates of glycolysis allow glycolytic intermediates to be channelled towards one-carbon metabolism to produce NADPH (reduced nicotinamide adenine dinucleotide phosphate) and nucleotides, enabling rapid biosynthesis for growth and proliferation [17]. This mechanism has been postulated to account for the Warburg effect, or aerobic glycolysis, by which proliferating cancer cells metabolise glucose via glycolysis even in the presence of oxygen [17,18].

The rate of glycolysis depends not only on the availability and uptake of glucose, but also on the kinetics of the metabolic enzymes that catalyse several rate-limiting steps along the pathway. Among these enzymes, hexokinase (HK), phosphofructokinase (PFK), and pyruvate kinase (PK) are allosterically regulated and have important roles in regulating glycolytic flux [19,20,21,22]. The initial switch that upregulates glycolysis in hypoxia has been traditionally attributed to the allosteric control of glycolytic enzymes by ATP [23]. This is followed by an important longer-term adaptive mechanism through HIF-1-dependent upregulation of the synthesis of glucose transporters and glycolytic enzymes [24,25,26,27]. This adaptive mechanism, which promotes survival of normal cells during hypoxia, is frequently hijacked by cancer cells, which then respire by aerobic glycolysis—the Warburg effect [18,28].

Although in normal cells HIF-1 signalling occupies a central role in orchestrating the adaptive response of glycolysis to hypoxia, loss of HIF-1 in cancer cells and solid tumours paradoxically does not perturb the glycolytic phenotype [15,16,29]. This suggests that there are HIF-1-independent cellular and molecular mechanisms that maintain glycolysis under hypoxic conditions.

In glycolysis, the biochemical reactions catalyzed by the rate-limiting enzymes—HK, PFK, and pyruvate kinase (PK)—are virtually irreversible and represent potential sites for control of glycolytic flux. To meet the changing metabolic demands of the cell, the activities of these enzymes can be regulated by reversible binding of allosteric effectors (e.g., ATP) or by covalent modification (e.g., phosphorylation), both of which usually occur on a much shorter timescale than HIF-induced transcriptional regulation. In a hypoxic normal cell, oxidative phosphorylation is inhibited, resulting in a decreased level of ATP and thus a decreased ATP/AMP (adenosine monophosphate) ratio. This reduces allosteric inhibition of ATP on PFK, the enzyme that catalyses the conversion of fructose-6-phosphate plus ATP to fructose 1,6-bisphosphate and ADP, thus promoting flux through the glycolytic pathway. The lower ATP/AMP ratio also reduces the allosteric inhibition of ATP on PK, causing more pyruvate to be produced from phosphoenolpyruvate; the increased fructose-1,6-bisphosphate also feeds-forward to activate PK activity, thus further increasing glycolytic flux. Together with transcriptional upregulation of glucose transporters and glycolytic enzymes induced by the HIF-1 transcription factor, these biochemical responses result in increased glycolytic flux, which maintains cellular ATP production under hypoxia.

## 3. HIF-1 Signaling & Control of Metabolism

The HIF-1 signalling pathway has been extensively reviewed elsewhere [2,3,30]. In this section, we will briefly recapitulate the salient features of this pathway and how it relates to energy metabolism.

### 3.1. Activation of HIF-1

**Figure 1:**
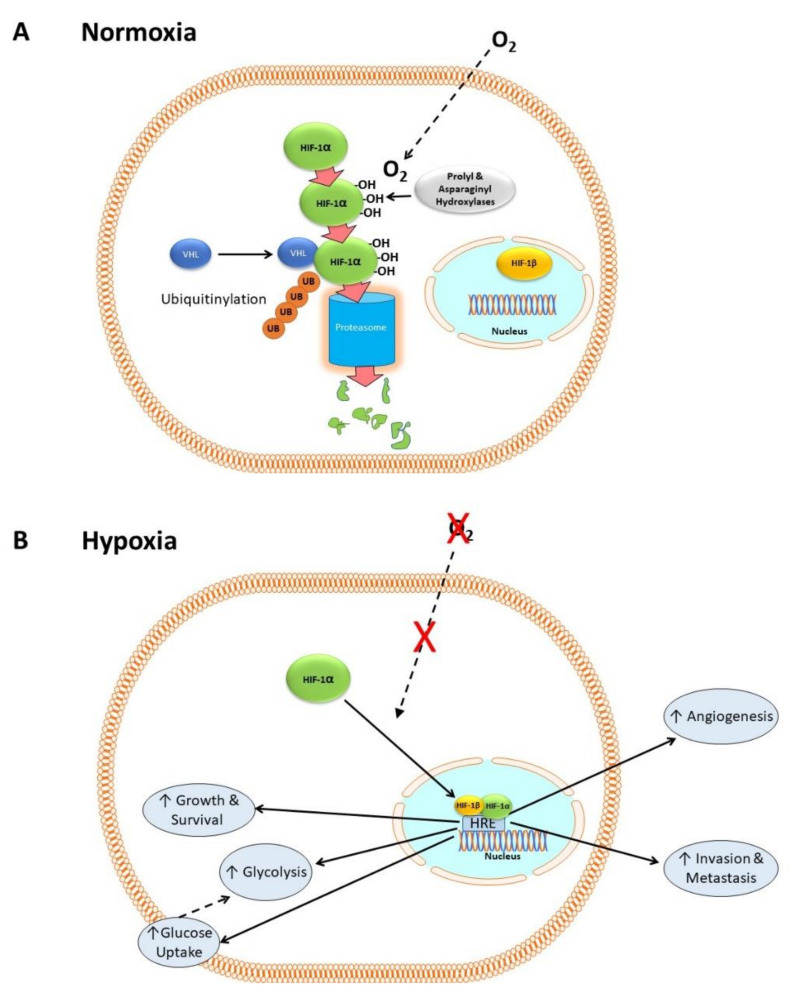
HIF-1 in normoxia and hypoxia. (**A**) In normoxia, HIF-1α is hydroxylated by prolyl and asparaginyl hydroxylases in the cytoplasm in an oxygen-dependent manner. It then forms a complex with VHL and other proteins, allowing it to be ubiquitnylated and degraded by proteosomes. (**B**) In hypoxia, the lack of oxygen-dependent hydroxylation and proteasomal degradation of HIF-1α causes it to accumulate. It then translocates to the nucleus and complexes with HIF-1β to form a transcription factor complex that binds to hypoxia response elements (HRE) within the promoter regions of target genes involved in cell proliferation, metastasis, glycolysis, angiogenesis, growth, and survival.

HIF-1 consists of two subunits: HIF-1α, which is cytosolic, and HIF-1β, which is constitutively expressed in the nucleus [31]. In the presence of dissolved oxygen (i.e., in normoxia) prolyl and asparaginyl hydroxylases catalyze the hydroxylation of HIF-1α [32,33,34], which then complexes with the Von Hippel–Lindau factor (VHL) and other proteins [35]. This condition allows HIF-1α to be ubiquitinylated, which in turn flags it for degradation by proteasomes [35,36,37,38]. Figure 1A shows a simplified version of this mechanism, neglecting numerous co-factors that are not relevant to the present discussion.

In hypoxia, there is no oxygen substrate for the hydroxylases, so HIF-1α accumulates, translocates to the nucleus and complexes with its co-factor HIF-1β. The HIF-1α/HIF-1β complex binds to hypoxia response elements (HRE), a transcription factor binding site within the promoter regions of target genes, activating the transcription of hundreds of genes including those for cell proliferation [39], metastasis [40,41,42,43], glycolysis [26,27], pH regulation [44], and angiogenesis [45]. Figure 1B shows a simplified outline of these mechanisms. HIF-1 is activated in many cancers and its activation correlates with poor outcome [7].

### 3.2. Control of Metabolism by Activated HIF-1

**Figure 2:**
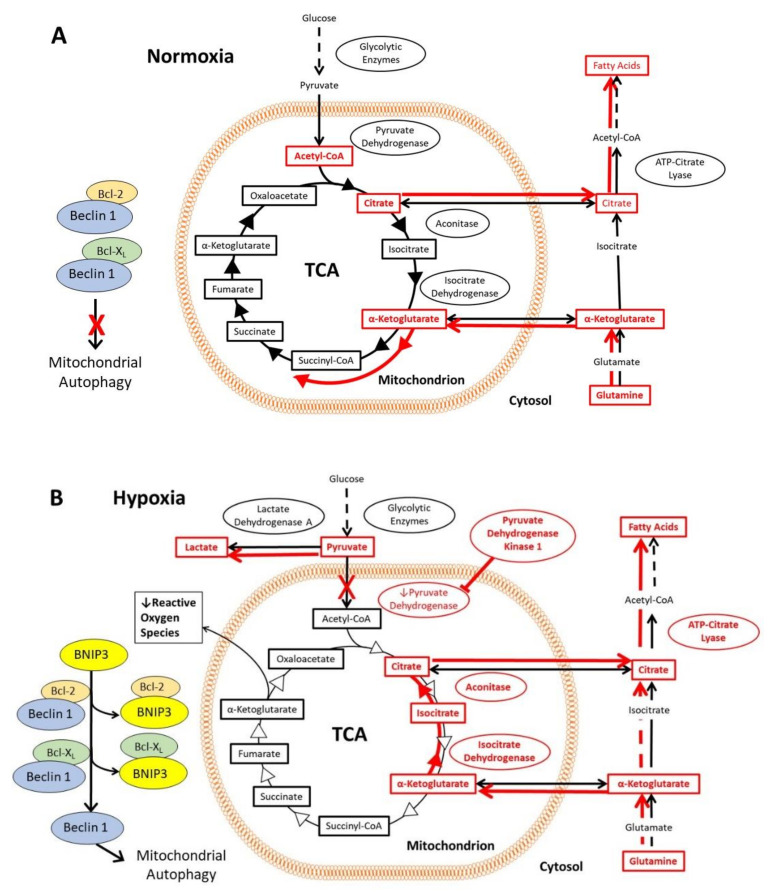
Effects of the HIF-1 activation on the tricarboxylic acid cycle and associated pathways, including glutamine and citrate metabolism, in hypoxia. The mechanisms are highlighted in red, and the red arrows indicate the direction of flux via the metabolic pathways under the prevailing oxygen tension. (**A**) In normoxia, pyruvate produced via glycolysis enters the mitochondria, where it is converted to acetyl-CoA and enters the tri-carboxylic acid cycle (TCA). Citrate from the TCA can be exported from the mitochondria into the cytosol, where it is converted to acetyl-CoA for fatty acid synthesis. Cytosolic glutamine is converted to α-ketoglutarate and transported into the mitochondria where it enters the TCA. A key regulator of autophagy, Beclin 1 is inhibited by Bcl-2 and Bcl-XL, thereby decreasing autophagy. (**B**) In hypoxia, HIF-1 activation results in increased flux through the glycolytic pathway, as well as accumulation of pyruvate and conversion into lactate. BNIP3 and BNIP3L (not shown in this figure) are expressed under the control of HIF-1, which then interact with Bcl-2 and Bcl-XL to liberate Beclin 1. Beclin 1 induces mitochondrial autophagy, reducing the formation of mitochondrial reactive oxygen species under hypoxia. Fatty acid synthesis is maintained by reductive carboxylation of glutamine, formation of citrate, and conversion of citrate into acetyl-CoA in the cytoplasm.

In normoxic cells, the glycolytic enzymes metabolize glucose to pyruvate, which then translocates into the mitochondrial matrix. There, it is converted to acetyl-CoA by pyruvate dehydrogenase and followed by entry into the tri-carboxylic acid cycle (TCA) [46]. The first step in the TCA is formation of citrate, some of which is shuttled across the mitochondrial membrane to the cytosol, where it is converted to acetyl-CoA by ATP-citrate lyase and subsequently used for fatty acid synthesis [47,48]. Conversely, cytosolic glutamine is converted to α-ketoglutarate and transported into the mitochondria, where it enters the TCA [46] (Figure 2A). In normoxia, the rate of autophagy is decreased, since Beclin 1, an important activator of autophagy, is inhibited through its interaction with Bcl-2 and Bcl-X_L_ proteins. In hypoxia, HIF-1 activates the transcription of genes encoding glucose transporters and glycolytic enzymes, which increases flux through the glycolytic pathway (Figure 2B). The expression of pyruvate dehydrogenase kinase 1 (PDK1) and other genes encoding isoforms of pyruvate dehydrogenase kinase results in phosphorylation and inactivation of pyruvate dehydrogenase [49,50]. Pyruvate therefore accumulates in the cytosol and is converted to lactate by lactate dehydrogenase A, the expression of which is also upregulated by HIF-1 [51]. Under hypoxia, mitochondria produce more reactive oxygen species (ROS). To counter the production of ROS, HIF-1 upregulates expression of BNIP3 and BNIP3L, which displaces Beclin 1 from Bcl-2 and Bcl-X_L_ [52,53]. The disinhibition of Beclin 1 induces mitochondrial autophagy, which in turn reduces the formation of ROS (Figure 2B). Another set of mechanisms maintains fatty acid synthesis despite the reduced flux of glucose to citrate (Figure 2B). Reductive carboxylation of glutamine generates α-ketoglutarate, much of which is taken into the mitochondrion and converted by isocitrate dehydrogenase 2 and aconitase 2 to citrate, which is exported again to the cytosol, where some of it is converted by the cytosolic isoenzymes isocitrate dehydrogenase 1 and aconitase 1 into acetyl CoA, and used for fatty acid synthesis [54,55]. Note that this part of the TCA runs backwards in this mechanism.

## 4. Glucose Metabolism in Cancer Cells in Deficient in HIF-1 under Hypoxia

Does the lack of HIF-1 impair the ability of cancer cells to adapt their metabolism under hypoxia? There is increasing evidence that cancer cells are capable of adapting their energy metabolism to survive under hypoxia in the absence of HIF-1. Such changes involve allosteric regulation of glycolytic enzymes, increased expression of glucose transporters, and recruitment of other metabolic pathways such as creatine, glutamine, and lipid metabolism.

### 4.1. Allosteric Regulation of Glycolytic Enzymes under Hypoxia

Experimental evidence for the involvement of allosteric control of glycolysis in the metabolic strategy of cancer cells to adapt to HIF-1 and HIF-2 deficiency was revealed in a series of studies on Hepa-1 c4 cells and tumors. Hepa-1 c4 cells are deficient in the HIF-1β subunit, which is an essential component of both the HIF-1 and HIF-2 active complexes, so they cannot form functional HIF-1 or HIF-2 (HIF-1/2) dimeric complexes or activate HIF-1/2-dependent gene transcription [13,14,15,16,29,56]. In addition, Knaup et al. (2017) found that Hepa WT tumours have no expression of HIF-2, which eliminates the possibility of any HIF-2-induced survival mechanisms [57]. This absence of both the HIF-1 and HIF-2 pathways makes Hepa c4 a preclinical model for cancers that have developed resistance to anticancer drugs that inhibit HIF-1 and/or HIF-2.

Despite lacking functional HIF-1/2 pathways, Hepa c4 cells were able to upregulate glycolysis to the same extent as their wild type (Hepa WT) counterparts when cultured under hypoxia [15,16,29]. In vivo, Hepa-1 c4 tumours grew slower than wild type tumours, and had significantly lower expression of several glycolytic enzymes, as well as lower levels of ATP (20% of the level in Hepa WT tumours) [15]. Nonetheless, assessment of glucose uptake using FDG-PET showed no difference between Hepa c4 and Hepa WT tumours, suggesting that glycolysis in c4 tumours was unaffected by the loss of the HIF-1 pathway [16]. Two non-HIF-1/2-dependent signalling pathways that may upregulate glycolysis (PI3K-Akt and c-Myc) were found to have either lower or similar expression in Hepa c4 (compared with Hepa WT) tumours, but c4 tumours were found to have a 4.5-fold higher AMP/ATP ratio as well as higher phosphofructokinase-1 (PFK-1) activity compared to their WT counterparts [16]. Phosphorylated AMP-kinase was also higher in the c4 tumours [16]. Collectively, these findings suggest that allosteric PFK-1 activation by small molecule metabolites could be a mechanism by which cancer cells adapt to HIF-1/2 deficiency under hypoxic conditions. 

### 4.2. Auxiliary Functions of Glucose Uptake & Creatine Metabolism in Hypoxia

A number of other studies have been performed on adaptations to hypoxia in HIF-1α deficient cancer cells. Note that unlike the HIF-1β deficient Hepa c4 model, these HIF-1α deficient models leave the HIF-2 pathway intact, allowing the possible development of HIF-2-dependent resistance mechanisms.

Using a combination of transcriptomic, proteomic, and metabolomic approaches, Valli et al. found that hypoxic HCT116 colorectal cancer cells were able to adapt to HIF-1α deletion via HIF-1-independent metabolic pathways [58]. There was an accumulation of glycolytic intermediates, with a block of conversion of fructose-1,6-bisphosphate (Fru-1,6-BP) to dihydroxyacetone phosphate (DHAP) at the level of aldolase [58] rather than the earlier step of conversion of fructose-6-phosphate (Fru-6P) to Fru-1,6BP by PFK observed in c4 cells and tumors [16]. This finding implicates aldolase as an important regulatory step in glycolysis under hypoxia and HIF-1 inhibition. The decoupling of glucose uptake from glycolysis in this instance suggests that glucose may be re-routed to fuel other metabolic processes, such as glycogen metabolism, the pentose phosphate pathway, and lipid metabolism. Additionally, even in the absence of HIF-1, and despite low levels of glycolytic enzymes and glycolytic flux, glucose uptake was maintained in HIF-1α knockout HCT116 cells by a specific isoform of the glucose transporter, GLUT14, whose expression was modulated by HIF-2α instead of HIF-1α [58]. 

The findings of Valli et al. also highlight a previously unappreciated role for creatine metabolism in buffering changes in the intracellular ATP pool. Creatine metabolism is an important system for energy buffering during periods of high ATP demand, such as in contracting skeletal muscle. The intracellular pool of ATP may be buffered by creatine kinases via the reversible reaction PCr + ADP ⇌ Cr + ATP, which also regenerates phosphocreatine (PCr) when ATP becomes abundant [59]. HIF-1α knockout HCT116 cells and spheroids were found to have higher levels of the cytoplasmic creatine kinase isoenzyme (creatine kinase B, CKB) but a lower level of the mitochondrial creatine kinase isoenzyme [58]. The cell membrane creatinine transporter SLC6A8 was found to be upregulated by hypoxia equally in both wild type and HIF-1α knockout HCT116 cells [54]. Targeting creatine metabolism by genetic silencing or pharmacological inhibition of CKB was found to greatly reduce the clonogenic potential and 3D cell spheroid growth of HIF-1 knockout cells under hypoxia compared to wild type controls [58]. This depletion of phosphocreatine reserves by CKB inhibition reduced the ability of the cancer cells to form metastatic outgrowths, a reflection of their ability to survive hypoxic environments. A number of hypoxic colorectal cancer cell lines have been found to release CKB into the extracellular space, where it catalyzed the production of PCr using extracellular ATP as the phosphate source [60]. This PCr was then imported into the cancer cells via SLC6A8 and generated ATP, thus enabling metastatic cancer cells to sustain their energetic requirements despite the physiologically hypoxic O_2_ levels in the normal liver [60]. The in vivo importance of this mechanism was demonstrated when mice inoculated with colorectal cancer cells and treated with cyclocreatine, an inhibitor of creatine metabolism, showed significant reduction in liver metastases [60]. 

Another interesting issue is that the upregulation of glucose transport by induction of GLUT14 that was observed by Valli et al. (2019) involved the activation of HIF-2 [58]. It is possible, therefore, that cancers could develop resistance to anti-HIF drugs targeted at HIF-1α by this and other HIF-2-dependent mechanisms. One solution would be the development of drugs targeted on HIF-1β rather than HIF-1α, since the activation of both HIF-1 and HIF-2 depends on complex formation with HIF-1β. However, HIF-1β is an unusual protein that also “moonlights” as the aryl hydrocarbon receptor nuclear translocator (ARNT), so it has effects on many bodily systems. ARNT inhibitors have been described, for instance, as an adjuvant for antibiotic therapy [61] and as an antifibrotic agent for kidney disease [62]. One would also expect such a pleiotropic agent to have off-target effects, and these have indeed been observed [63].

## 5. Glutamine Metabolism & Hypoxia

Glutamine metabolism has also been implicated in a HIF-independent hypoxia response. Glutamine, a nonessential amino acid, is commonly regarded as the second-most important metabolic fuel after glucose. It plays a central role in a variety of biological processes and is key in supporting cell proliferation [64,65]. The evidence for the necessity of glutamine for cancer progression is conflicted: some studies have reported that tumours can grow well without glutamine [66,67], while others showed that cancers are glutamine-addicted [68,69,70]. Furthermore, depending on the tissue of origin, cancer cells use various routes of glutamine metabolism. Some have been shown to require an exogenous glutamine supply for malignant proliferation [71,72], whereas others do not [73] and can synthesize it de novo [74]. It has been suggested that cells can be conditionally dependent on glutamine, depending on the growth conditions [75]. Interestingly, different cancer subtypes exhibit distinct patterns of glutamine utilisation: most basal breast cancer cells require glutamine supplementation in the media, whereas luminal breast cancers are less glutamine dependent [76]. Variation in glutamine fate has also been reported to be based on growth conditions: cancer cells metabolised glutamine differently when they were cultured as monolayers or spheroids [77] or when they were transplanted as tumours [78]. 

### Myc, Glutamine Metabolism & HIF-1 Deficiency

Myc is a transcription factor with pleiotropic effects, resulting in diverse cellular processes that promote tumour growth and progression. It has long been established that Myc activation upregulates glutamine metabolism [79,80], increasing glutamine flux by upregulating the glutamine transporter (ASCT2) and glutaminase-1, thus increasing glutamine conversion to glutamate [81].

Numerous studies have reported that malignant cells use glutaminolysis under hypoxic conditions [82,83]. In particular, glutaminase, a key enzyme converting glutamine to glutamate, was found to be increased in several cell types, including osteoblasts [84] and, via HIF-1, in colorectal cancer cells, which required glutaminase for hypoxia-induced migration and invasion in vitro, and tumour growth and metastatic colonisation in vivo [85]. The relationship between Myc and HIF is complex [86]. It has been shown that HIF-1α and HIF-2α have opposing effects on Myc; HIF-1α decreased Myc expression [87] while HIF-2α promoted c-Myc activity in various cell types [88]. Myc and HIF-2 have been shown to work together to negatively regulate p53 and reduce ROS levels in cancer stem cells [89]. Interestingly, a study in pancreatic ductal adenocarcinoma cells reported that genetic knockdown of HIF-2α decreased glutamine consumption. Decreased levels of HIF-2α did not influence glutaminase but contributed to a reduced expression of glutamate oxaloacetate transaminase 1, an enzyme responsible for conversion of glutamine-derived aspartate to oxaloacetate. This observation was related to the activation of the PI3K/TORC2 pathway [90]. Myc overexpression has been found to compensate for the lack of HIF-1 activity in hypoxic small cell lung cancer cells through upregulating glutaminolysis and lipogenesis. By studying two Myc-overexpressing SCLC cells that lack HIF-2 expression, Thoren et al. found that these cells did not require HIF activation and induced a hypoxic response by activating glutamine metabolism and de novo lipogenesis [91]. The decrease in HIF-2α expression in these cells correlated with an increase in expression of Myc genes, suggesting that Myc may act as a HIF-independent sensor of hypoxia [91]. Intriguingly, the genetic knockdown of HIF-1α expression had little to no effect on cell survival and growth under hypoxia, either in vitro or in vivo, and no effect on ATP levels [91]. In these HIF-1-deficient cells, glutamine was found to be critical and sufficient to produce the ATP required for cell growth, since blocking the conversion of glutamine to glutamate by inhibition of glutaminase caused cell death, whereas glucose withdrawal from the media did not interfere with cell viability [91]. Furthermore, those HIF-deficient hypoxic cells had higher expression of proteins involved in fatty acid metabolism, including ATP-citrate lyase, acetyl-CoA carboxylase and fatty acid synthase, while a query of a clinical SCLC databank showed a positive correlation between high expression of Myc and fatty acid synthase [91]. Increases in lipogenesis-related genes and glutamine consumption suggest that both glutamine and fatty acid metabolism have roles in Myc-driven, HIF-independent hypoxia responses.

## 6. Lipid Metabolism & Hypoxia

The links between hypoxia, HIFs, and lipid metabolism have been extensively reviewed elsewhere [92]. Hypoxia-mediated changes in lipid metabolism are essential to maintain tumor cell proliferation within hypoxic microenvironments, since proliferation requires the biosynthesis of new cell membrane and production of signaling molecules, both derived from fatty acids. Under hypoxic conditions, the TCA cycle is inhibited, and this blocks the conversion of glucose into citrate, a major source of cytoplasmic acetyl-CoA and fatty acid precursors. Hypoxic cancer cells therefore have to resort to alternative sources of fatty acids, such as scavenging exogenous fatty acids from the extracellular space and forming them into lipid droplets. This may partially explain the observation that cancer cells have a higher number of lipid droplets to store triacylglycerides and cholesterol derivatives compared to normal cells, suggesting an alteration of lipid metabolism towards a more lipogenic phenotype [93]. Alternatively, certain cancer cells may use carbon sources such as glutamine or acetate to compensate for the reduction in acetyl-CoA synthesis from glucose [94,95]. 

In many cancer types, the silencing of HIFs under hypoxia, or interference with the expression of their target genes required for lipid accumulation, results in reduction of proliferation and chemoresistance. In the study by Valli et al. discussed earlier, hypoxia was also found to induce changes in several lipid metabolites by both HIF-dependent and HIF-independent mechanisms [96]. Enzymatic steps in fatty acid synthesis and the Kennedy pathway were altered in a HIF-1α-dependent manner, whereas palmitate, stearate, phospholipase D3 (PLD3), and platelet-activating factor homologous C16 (PAFC16) were regulated in a HIF-1α-independent manner [96]. This HIF-1α-independent metabolic signature involved HIF-2α upregulation in association with impaired fatty acid (FA) β-oxidation, increased cellular lipid storage, and increased cell proliferation, while palmitate and stearate were observed to accumulate in a HIF-1α-independent manner [96]. Accumulation of fatty acids provides metabolic precursors for β-oxidation in proliferating cells recovering after re-oxygenation, and fatty acids can also act as a free radical buffer via lipid peroxidation [96]. Platelet activating factor (PAF) is a potent, inflammatory lipid mediator that stimulates the release of nitric oxide, which in turn increases vascular permeability and vasodilatation. The C-16 homolog of PAF was found to accumulate in hypoxia in a HIF-1α-/HIF-2α-independent manner due to reduced PAFC16 catabolism from lowered levels of PLD3 [96]. These findings suggest that lipogenesis is an important metabolic adaptation for cancer cells lacking HIF-1α under hypoxic conditions.

## 7. Potential Alternative Metabolic Adaptations to Survive Hypoxia in HIF-1 Deficiency

In this section, we will explore several possible alternative mechanisms, in addition to allosteric regulation of glycolytic enzymes, that could be employed by cancer cells to adapt to hypoxia in the face of HIF-1 deficiency. Insights into novel HIF-1-independent mechanisms regulating glycolysis under hypoxia that emerged in the last decade include epigenetic reprogramming [97], post-translation modifications [98], spatial reorganization of glycolytic enzymes [98,99] and the PI3K-Akt-mTOR signalling pathway [100]. We will also explore the links between glutamine metabolism and 2-hydroxyglutarate as a potential hypoxia bypass mechanism in HIF-1 deficiency.

### 7.1. Allosteric Regulation of Glycolysis by PFK-2/FBPase-2

Besides allosteric regulation of PFK-1 by ATP (discussed earlier in Section 3.1), another important allosteric activator of PFK-1 is fructose 2,6-bisphosphate (Fru-2,6-P2), a metabolite synthesised from fructose 6-phosphate in a reaction catalysed by 6-phosphofructo-2-kinase/fructose 2,6-bisphosphatases (PFK-2/FBPase-2). While PFK-1 can be inhibited by ATP to limit glycolytic flux under aerobic conditions (the Pasteur effect), allosteric activation of PFK-1 by Fru-2,6-P2 relieves this inhibition. However, there is thus far no experimental evidence to suggest that this mechanism might be employed by cancer cells to overcome HIF-1 deficiency. The PFK-2/FBPase-2 family comprises several enzyme isoforms, PFKFB1, PFKFB2, PFKFB3 and PFKFB4, all of which are induced by HIF-1 under hypoxic conditions [101]. Of these isoforms, the expression of PFKFB2 has been found to be transcriptionally upregulated by androgen receptor (AR) signaling in prostate cancer cells, involving downstream activation of the calcium-calmodulin/dependent protein kinase II (CAMKII)-AMPK signaling pathway, and also by direct recruitment of the ligand-activated androgen receptor to the PFKFB2 promoter [102]. Abrogation of PFKFB2 expression in prostate cancer cells reduced both glycolysis and lipogenesis [103]. Moreover, changes in hypoxia/reoxygenation have previously been found to stimulate androgen receptor trans-activation and sensitization in prostate cancer cells that is independent of HIF-1 signaling [104]. Therefore, it is compelling to speculate that transcriptional upregulation of PFKFB2 by androgen receptor signaling in hypoxia may represent a potential mechanism by which prostate cancer cells could allosterically upregulate glycolysis in the absence of HIF-1.

### 7.2. Epigenetic Regulation of Glycolysis under Hypoxia

Epigenetic reprogramming is another important means by which hypoxia regulates cell metabolism independently of HIF-1. Epigenetics refers to heritable molecular alterations in the chromatin that regulates gene expression without altering the sequence of DNA bases in the genes [105]. These alterations, which include DNA histone methylation and other modifications, can be strongly influenced by cellular milieux such as hypoxia [106].

Histone methylation of genes is regulated by histone methyltransferases, which add methyl groups to arginine and lysine residues, and by demethylases (lysine demethylase—KDM), which remove these methyl groups [105]. KDM enzymes are 2-oxoglutarate-dependent dioxygenases which are dependent on Fe^2+^ and oxygen in order to remove methyl groups by hydroxylation; they include the Jumonji domain histone demethylases (JHDMs) which utilize that mechanism to hydroxylate and remove the methyl groups from lysine residues of modified histones [107].

Hypoxia increases bulk histone methylation either through inhibition of JHDMs or increased methyltransferase activity [108]. The earliest study demonstrating a link between hypoxia and the epigenetic regulation of specific HIF-related genes found that the expression of three Jumonji domain histone demethylase (JHDM) genes, JMJD1A (KDM3A), JMJD2B (KDM4B), and JARID1B (KDM5B), was HIF-1α-dependent [97]. Among these, JMJD1A was found to regulate a subset of hypoxia-induced genes, ADM, EDN1, HMOX1, and GDF15, and was important for the growth of tumour xenografts in a hypoxic environment [97]. JMJD1A increases or maintains expression of certain genes under hypoxia by reducing H3K9 demethylation of specific hypoxia-responsive promoters, including ADM, EDN1, HMOX1, and GDF15, resulting in increased expression of these genes and favouring tumour cell growth [97]. JMJD1A was found to co-activate with HIF-1α to induce the expression of genes involved in glucose metabolism, including GLUT1, HK2, PGK1, PGM, LDHA and MCT4, by demethylating H3K9me2 on their promoter regions [109]. Genetic knockdown of JMJD1A reduced the expression of these genes, resulting in decreased urinary bladder cancer cell proliferation, colony formation and xenograft tumour growth [109]. However, it remains unknown why JMJD1A is targeted to some hypoxia-inducible target genes but not to others.

Hypoxia has been found to directly modulate the enzymatic activity of histone demethylases independently of HIF, with important implications for the control of metabolism in cancers. Two members of the Jumonji domain KDM enzymes, KDM5A and KDM 6A, were recently implicated as oxygen sensors that become inactivated during hypoxia, causing rapid histone hypermethylation [110,111]. These rapid histone hypermethylations occurred independently of HIF-1 and of other known hypoxia-inducible inhibitors of KDM activity, such as reactive oxygen species and 2-hydroxyglutarate [110,111]. Overexpressed in several different types of human cancers [112], KDM5A has been reported to control differentiation and mitochondrial metabolism by directly repressing metabolic regulatory genes. Loss of the *KDM5A* gene in pRb-deficient murine embryonic fibroblasts restored differentiation by increasing mitochondrial respiration [113]. KDM5A was also found to suppress the transcription of mitochondrial pyruvate carrier-1 (MPC-1)—a mediator of pyruvate import into the mitochondrion—by H3K4 trimethylation, thereby reducing mitochondrial metabolism [114].

In contrast to KDM5A, the effect of KDM6A on metabolism is less clear. Loss or inactivation of KDM6A has been found to promote several malignancies, with genomic analyses revealing loss of the *UTX* gene coding for KDM6A in various cancers [115,116,117,118]. In a *Kras*^G12D^-driven pancreatic intraepithelial neoplasia mouse model, tumorigenesis was suppressed in mice with a functional *KDM6A* gene expression, whereas deletion of *KDM6A* gene alone in female mice or concomitant deletion of *KDM6A* and *UTY* (*KDM6A* gene homolog on the Y-chromosome) genes in male mice induced aggressive, poorly differentiated squamous-like tumours via activation of diverse signalling pathways associated with the epithelial-mesenchymal transition (EMT), inducing proliferation, inflammation, and hypoxia [119]. Furthermore, in an oncogenic *Kras*^G12D^-induced lung cancer mouse, deletion of *KDM6A* gene accelerated disease progression and increased tumour burden [120]. In contrast, high KDM6A and KDM6B demethylase activity with correspondingly lower levels of H3K27 trimethylation were observed in posterior fossa A ependymomas, a rare paediatric tumour that strictly depends on hypoxia for survival and exhibits high levels of glycolysis, non-oxidative pentose phosphate and polyamine metabolism [121]. The high demethylase activity in this type of tumour was in turn dependent on a high α-ketoglutarate/succinate ratio generated by glutaminolysis and reduced TCA flux [121]. Insights from the field of induced pluripotent stem cells (IPSCs) suggest that KDM6A may improve IPSC reprogramming efficiency, in part by demethylation of H3K27, on metabolism-related genes, resulting in increased glycolysis and amino acid uptake via the PI3K-Akt-mTOR pathway, with only minimal contribution from the HIF-1 signalling pathway [122]. It is evident that we are only beginning to understand the link between KDM enzymes, hypoxia, metabolism and epigenetics, and it is likely that there are several more layers of complexity in their interactions.

### 7.3. Post-Translational Modifications of Glycolytic Enzymes under Hypoxia

Post-translational modification of glycolytic enzymes is an important mode of regulation in glycolysis. SUMOylation is a reversible posttranslational modification pathway catalysing the conjugation of small ubiquitin-related modifier (SUMO) proteins to lysine residues of distinct target proteins, thereby affecting a multitude of key processes in a highly dynamic manner. The link between SUMOylation and hypoxia has been extensively reviewed elsewhere [123]. Increased global SUMO conjugation upon hypoxia was initially reported as a result of increased SUMO1 expression in hypoxic cultured T84 colon cells, and in the brains and hearts of mice exposed to 10% oxygen [124,125]. SUMOylation has been found to impact key elements of the HIF signalling pathways, including HIF-1, HIF-2, ARNT, PHD3, FIH, CBP/p300, and pVHL [123]. Besides directly interacting with regulators of HIF signalling, SUMOylation is known to modulate the function of many other signalling pathways that indirectly impact hypoxia signalling, such as the Ras/MAPK, PI3K/Akt, and NF-κB signalling pathways, as well as downstream targets such as VEGF-induced angiogenesis, GLUT1, and GLUT4 [123]. 

In the context of cellular metabolism, Agbor et al. first observed that the induction of hypoxia-induced glycolysis was retained by cells when gene transcription or protein synthesis were inhibited, suggesting the involvement of additional post-translational mechanisms [98]. Using mass spectrometric analysis on *Saccharomyces cerevisiae* cells, they found that hypoxia induced post-translational protein modification of the key glycolytic enzymes glyceraldehyde 3-phosphate dehydrogenase and phosphoglycerate kinase by SUMO-1 [98]. Overexpression of SUMO-1 in cancer cells resulted in increased hypoxia-induced glycolysis and resistance to hypoxia-dependent ATP depletion [98]. Intriguingly, these cells also demonstrated focal clustering of glycolytic enzymes in response to hypoxia, suggesting that SUMOylation may promote spatial reorganisation of the glycolytic pathway [98], another mechanism by which glycolysis can be regulated that is discussed below. In contrast, SUMO-specific protease 2 (SENP2), a de-SUMOylating enzyme, was found to negatively regulate aerobic glycolysis in MCF7 and MEF cells [126]. Overexpression of SENP2 reduced glucose uptake and lactate production and increased ATP levels in MCF7 cells, whereas knockout of this enzyme in murine embryonic fibroblast cells produced the opposite effect [126]. This phenotype was rescued by an AKT inhibitor, suggesting that SENP2 represses glycolysis in part through inhibition of AKT phosphorylation and PI3K-Akt signalling [126].

### 7.4. Spatial Reorganisation of Glycolytic Enzymes under Hypoxia

The traditional approach to biochemistry views metabolic pathways as a series of chemical reactions catalysed by randomly arranged enzyme molecules. However, in reality, enzymes do not always operate in isolation but instead frequently localize together to form molecular machines or protein complexes that integrate enzymatic activities. These supramolecular complexes increase enzymatic efficiency by substrate and product channelling between enzymes [127].

Spatial organization of metabolic multienzyme complexes has long been hypothesized as a means to regulate metabolism. The first study to demonstrate a link between hypoxia and spatial organisation of glycolytic enzymes was reported by Jin et al., who found that hypoxia resulted in the concentration of glycolytic enzymes, including the Pfk2p subunit of the rate-limiting PFK-1, into foci of membraneless granules termed ‘G bodies’ in both *Saccharomyces cerevisiae* and human hepatocarcinoma cells [99]. In yeast, the presence of G bodies correlates with accelerated glucose consumption, and impairing G body formation leads to the accumulation of upstream glycolytic metabolites [99]. Intriguingly, Snf1p—the yeast ortholog of AMPK—and RNA were required for the formation of G bodies [99]. Hypoxia-induced formation of G bodies in yeast comes about through RNA binding to glycolytic enzymes, forming ribonucleoprotein granules, which enhances the rate of glycolysis [128]. 

In *C. elegans,* hypoxia was found to rapidly induce the formation of foci-containing glycolytic enzymes at presynaptic sites in neurons in order to meet the energetic requirements of synaptic signalling [129]. Furthermore, various human cancer cell lines—but not normal cells—have been found to form small aggregates of glycolytic enzymes (comprising liver-type PFK-1, FBPase, pyruvate kinase M2, and phosphoenolpyruvate carboxykinase-1) even in the presence of oxygen [130]. Intriguingly, the formation of medium- and large-sized clusters at the single-cell level was found to correspond to the metabolic shunts of glucose flux into the pentose phosphate pathway and serine biosynthesis, respectively [130]. A number of mechanisms have been postulated to explain how glycolytic flux is enhanced by the formation of G bodies. These include decreased substrate inhibition by phase separation, substrate channelling by concentration of intermediate metabolites, and enhanced translation of glycolytic enzymes by concentrating glycolytic enzymes with their cognate mRNAs. Overall, these findings suggest that the formation of G bodies is a highly conserved, adaptive response that increases glycolytic output during hypoxia or tumorigenesis, although the mechanisms by which hypoxia is sensed by regulators of the G bodies remain unknown.

### 7.5. PI3K-Akt-mTOR Signalling & Glycolysis in Hypoxia

PI3K-Akt-mTOR is a highly conserved intracellular signalling pathway that is important in regulating the cell cycle in response to extracellular signals. The phosphoinositide-3-kinases are a large family of lipid signalling enzymes that phosphorylate the inositol ring 3′-OH group in inositol phospholipids of the plasma membrane [131]. This signalling pathway begins with the generation of phosphatidylinositol (3,4,5) trisphosphate [PI(3,4,5)P] from phosphatidylinositol (4,5) bisphosphate [PI(4,5)P2] by the action of phosphoinositide-3-kinase (PI3K) and the subsequent activation of Akt (protein kinase B) and its downstream signalling cascades (e.g., mTOR). PI3K-Akt-mTOR is implicated in diverse cellular processes, including metabolism, inflammation, cell survival, and cancer progression [131]. With regards to metabolism, the activation of the PI3K-Akt pathway upregulates glucose transporter expression, increases glucose uptake, and stimulates PFK activity [100,132].

While the effect of the PI3K/Akt signalling pathway on glycolysis has been shown to be mediated in part via its regulation of HIF-1α expression and transcriptional activity in hypoxia [133,134], several lines of evidence have shown that its effects on glucose metabolism are also mediated via HIF-independent mechanisms. Under normoxia and in response to cytokine or growth factor stimulation, PI3K/Akt activation is able to regulate glycolysis via direct regulation of GLUT1 activity and localization [135] and by increasing the expression of the rate-limiting enzymes hexokinase-2 and PFK [100,136] without activating mitochondrial oxidative phosphorylation [137]. Interestingly, in hepatoma cells lacking HIF-1 signalling, PI3K-Akt signalling has been reported to promote tumour growth via increased VEGF and vascularisation, although that study did not measure glycolysis in these tumours [134]. In contrast, HIF-1/2-deficient Hepa c4 cells and tumours showed no difference in the level of Akt phosphorylation compared to their wild type counterpart [16]. The importance of PI3K/Akt signalling on glycolysis under hypoxic conditions in HIF-1-deficient tumours therefore remains unclear. 

### 7.6. Glutamine & 2-Hydroxyglutarate Metabolism

Glutamine has been reported as a primary carbon-source for the biosynthesis of 2-hydroxyglutarate (2-HG) [72,138], an oncometabolite proposed as a factor enabling progression of malignancies [139,140]. Elevated levels of 2-HG have been reported in cancers of blood (acute myelogenous leukaemia and some T-cell acute lymphoblastic leukemias), brain (low-grade gliomas, secondary glioblastomas) and also chondrosarcomas and cholangiocarcinomas [141,142,143,144,145].

The mechanism of 2-hydroxyglutarate (2-HG) generation is still under investigation, and various routes of its production have been proposed. It has been shown that 2-HG accumulates as a result of a gain-of-function mutation in the isocitrate dehydrogenase (IDH1/2) enzyme [146,147], a frequently mutated metabolic gene in human cancer. In cells with this defect, IDH loses affinity for its main substrate—isocitrate—and instead converts α-ketoglutarate (α-KG) into 2-hydroxyglutarate (2-HG). This leads to further dysregulation of TCA flux and redox status [148,149,150]. 2-HG has been implicated in the dysregulation of gene expression, as it can inhibit α-KG-dependent chromatin-modifying enzymes: dioxygenases and demethylases [151]. This oncometabolite is believed to act as an antagonist of α-KG, and by competitively inhibiting the activity of α-KG/Fe(II)-dependent dioxygenases, it hinders differentiation and promotes cell transformation [152]. Furthermore, 2-HG can cause histone hypermethylation [153,154]. Interestingly, KDM6A histone demethylase has been shown to influence cell differentiation in a HIF-independent manner [110].

Moreover, 2-HG has been shown to cause the reprogramming of the mTOR and HIF pathways [140,155,156], although the exact mechanism of interaction between 2-HG and HIF remains unknown. It has been speculated that since α-KG is a substrate for prolyl hydroxylases, synthesis of 2-HG and the resulting diminishing of α-KG levels will tend to block the HIF-1α degradation pathway, thus triggering the expression of HIF target genes and initiation of the hypoxia response [152,153,154]. However, Oldham et al. argue against this mechanism by showing that neither silencing nor overexpression of 2-HG impacts HIF stabilisation [157]. Additionally, because of the complexity of HIF regulation, low α-KG amounts may induce a HIF response via a number of other mechanisms that could include intracellular signalling cascades [AMPK, p38 mitogen-activated protein kinases (p38MAPK), AKT, ROS generation and microRNAs [158,159,160]. Therefore, it is plausible that increased levels of 2-HG may enable a switch to an entirely HIF-independent mechanism.

Furthermore, it has been speculated that distinct enantiomers of 2-HG are produced in response to different stimuli. Indeed, it has been reported that in response to hypoxia, mammalian cells synthesize the L-2-HG enantiomer via the reduction reaction of α-KG [161], and elevated amounts of that enantiomer were observed in VHL-mutated renal cell carcinoma tissues [162].

Accumulation of 2-HG has been confirmed in various normal and immortalised cell types, and increased amounts of L-2-HG were shown to be produced in response to mitochondrial dysfunction in hypoxia. This process was shown to be independent of HIF activation and may constitute a common hypoxia response mechanism [157]. Others have shown that D-2-HG inhibits electron transfer complexes I, IV, and V, and thus interferes with mitochondrial function [163]. Mitochondrial 2-HG dehydrogenase, which normally oxidizes 2-HG back to α-KG, may play a key role in this process; its decreased oxidative activity and/or increased reductive activity may be responsible for elevated amounts of 2-HG, as shown in HEK-293 cells [164]. Therefore, it remains unclear whether malfunctioning mitochondria are the cause or the result of 2-HG accumulation, or whether this effect relates to different actions of the enantiomeric forms L-2-HG and D-2-HG. Indeed, Oldham et al. suggested that hypoxia caused an increase in L-2-HG, but not D-2-HG levels, and that L-2-HG may come from different carbon sources, i.e., glycolysis or glutaminolysis, depending on cell type [157]. Further studies are needed to clarify the role of 2-HG enantiomers in signalling during hypoxia. An increased growth of astrocytes was observed in conjunction with D-2-HG accumulation but not with accumulation of the L enantiomer. Only D-2-HG was able to stimulate prolyl hydroxylases and thus impair HIF expression and enhance proliferation of astrocytes [165]. Moreover, D-2-HG caused destabilisation of HIF-1 in T cells [156]. However, a different study found that L-2-HG stabilised HIF-1α by inhibiting prolyl hydroxylases [166]. Moreover, D-2-HG has been linked to the activation of mTOR and nuclear factor kappa B (NF-κB) signalling [155,167]. Furthermore, studies with a knock-in, single-codon mutation in IDH1 in HCT116 colorectal adenocarcinoma cells showed that production of the D enantiomer of 2-HG altered the metabolism of those cells in two ways. Firstly, cells preferentially used glutamine rather than glucose as a carbon source for lipogenesis. Secondly, palmitate derived from glutamine increased in hypoxia but not in normoxia [168].

However, mutations in IDH are not the only source of 2-HG production. Some enzymes are capable of synthesizing 2-HG in promiscuous reactions, i.e., those which accompany specific, primary reactions of a given enzyme and produce different products under certain conditions, for example under acidic or hypoxic stress [169]. It was recently demonstrated that 2-HG can be produced via lactate dehydrogenase A (LDHA) in hypoxic conditions [157,161], and that 3-phosphoglycerate dehydrogenase (PHGDH) and malate dehydrogenase (MDH) are also capable of promiscuously producing 2-HG. It has also been shown that low pH resulting from acidosis in hypoxia affects substrate affinity of LDH and to a lesser extent MDH, resulting in stimulation of L-2-HG production [161,166]. Interestingly, it has been shown that mutated IDH1 impacts glycolysis in gliomas by downregulating LDHA [170], thus suggesting an inversely proportional correlation between those two enzymes. The relationship between LDHA and IDH1 and the impact of those enzymes on 2-HG enantiomer production need further clarification. 

The relationship between raising levels of 2-HG and HIF expression needs further clarification: some reports suggest that enhanced expression of 2-HG causes HIF stabilisation (L-2-HG, [166]) or destabilisation (D-2-HG, [156]), while others show that a hypoxic response occurs via HIF-independent signalling [157]. The different enantiomeric forms of 2-HG may thus induce different responses to hypoxia, including a HIF-independent one and an altogether different tumour phenotype. As already mentioned, there are other reactions than that of mutated IDH that can contribute to 2-HG accumulation. Indeed, increased amounts of 2-HG were observed in breast tumours where IDH mutations were not detected [171,172]. In those samples, 2-HG was found in millimolar concentrations, comparable with the concentrations occurring in *IDH1*-mutated cells [173]. Moreover, it has been shown that 2-HG was formed in mitochondria of breast cancer cells by a wild-type IDH2 in a so-called truncated TCA cycle, and was associated with Myc. Myc has been proposed to augment IDH2-driven reductive carboxylation of α-KG into isocitrate and reduction of 2-OG into 2-HG [174]. Interestingly, it has also been shown that IDH1 expression can depend on Myc [175].

Additionally, D-2-HG can also be produced by the mitochondrial alcohol dehydrogenase, iron-containing 1 enzyme (ADHFE1), and this process has been related to Myc oncogene expression [176]. In human breast tumours, ADHFE1 promoted production of *D-2*-HG via reductive glutamine metabolism, which also contributed to formation of mitochondrial ROS. Significant upregulation of the D enantiomer was observed in hypoxia, though HIF expression was not measured [177]. Interestingly, a competition between ADHFE1 and IDH2 for 2-HG production has recently been proposed [178]. The above studies suggest the involvement of Myc in the production of 2-HG.

### 7.7. The AMPK Pathway & Metabolic Adaptation

AMPK is a cellular energy sensor that enables a switch from anabolism to catabolism to facilitate cell survival in stressed conditions [179]. Its expression is induced by low substrate availability and/or by low oxygen tension. It is widely accepted that AMPK plays a major role in cellular adaptation to hypoxic stress [180], and its enhanced expression is indispensable for protecting cancer cells from hypoxic insult, in part by influencing metabolism; however, the exact mechanism is still under investigation. There is a significant amount of conflicting evidence in the literature regarding the role of AMPK in cancer [181].

Firstly, AMPK is considered to be a member of a tumour suppressor pathway, as it downregulates mTOR [182] and activates the tumour suppressor p53 [183]. Inhibition of its upstream coactivator LKB1 enables proliferation of melanoma cells [184]. Moreover, AMPK has been shown to negatively regulate the Warburg effect in vivo, and the loss of the AMPK-α subunit accelerated Myc-induced development of lymphoma [185]. However, others have demonstrated a pro-oncogenic role of AMPK in murine and human cells [186,187]. Tumour growth was supported by AMPK facilitation of NADPH homeostasis in breast cancer cells [188] or by AMPK-governed enhanced glycolytic flux in pancreatic cancer cells [187]. Deficiency in LKB1 caused a decrease in AMPK expression and prevented oncogenic transformation [189]. In other models, such as lung cancers, continued proliferation despite decreased LKB1 expression was attributed to activation of AMPK by calcium signalling [188,190].

Secondly, a shift in the AMP/ATP ratio is a known mechanism for activating AMPK [191,192]. However, others have shown that activation of AMPK in response to low oxygen tensions is independent of the AMP/ATP ratio but is governed by an increase in mitochondrial ROS [193]. A Ca2^+^-dependent activation of AMPK by CAMKK2 under hypoxia has also been demonstrated [194,195].

Finally, AMPK is known to act in concert with HIF [196], but there are also reports that it can act independently. Some studies even suggested an antagonistic mechanism where AMPK-deficient cells stabilised HIF and increased lactate production [185]. It was also shown that AMPK regulated HIF expression by maintaining high α-KG levels and thus stimulating prolyl hydroxylases [197]. Furthermore, AMPK has been shown to secure cellular energy balance under hypoxia by promoting autophagy independently of the HIF pathway [198].

Another mode of HIF-independent activation of AMPK was attributed to the loss of fumarate hydratase, where a receptor-mediated mechanism rather than metabolic activity of accumulated fumarate was a casual factor [186]. The authors reported that fumarate dehydratase-defective cells required activation of AMPK, but not of HIF-1/2, to avoid apoptosis. Interestingly, fumarate is linked with glutamine metabolism, as it is generated from α-KG. As mentioned before, AMPK is considered a tumour suppressor, since it inhibits the mTOR pathway. In C. elegans, both α-KG [199] and α-KG-derived 2-HG [200] suppressed growth by inhibiting ATP synthase, activating AMPK and decreasing signalling via the mTOR pathway. 

#### AMPK-PGC1a Signalling Axis

AMPK is known to activate PGC1α [201], a transcriptional coactivator that is regarded as a key regulator of mitochondrial metabolism. The interplay between HIF and PGC1α is not yet well-understood; however, evidence to date suggests a negative correlation between those two factors. Downregulation of PGC1α is linked to HIF-1α stabilization [202]. In human hepatoma cells, HIF-1α has been found to cause repression of Myc, resulting in decreased expression of *PGC-1*β, which led to the inhibition of mitochondrial fatty acid β-oxidation [203]. Interestingly, overexpression of PGC-1α in normoxic skeletal muscle cells contributes to increased oxygen consumption, followed by stabilisation of HIF-1α [204].

It has been reported that cells can increase expression of PGC-1α in response to low oxygen, that this increase is not driven by HIF-1 or HIF-2 [205], and that the expression of VEGF and resulting angiogenesis is induced by PGC-1α even in the absence of functioning HIF [206]. It has also been postulated that PGC-1α expression can be independent of HIF-activating prolyl hydroxylases, as small-molecule inhibitors of these enzymes did not influence the amount of PGC-1α protein [206].

In skeletal muscle cells, PGC-1α required ERR-α for the HIF-independent upregulation of VEGF. ERR-α (oestrogen-related receptor-α) is involved in the control of cell bioenergetics and is known to support tumour proliferation, angiogenesis and metastasis [207]. Increasing evidence suggests that the PGC-1α/ERRα axis plays a key role in cancer development and therapy resistance. The interplay between those two factors acts as a sensor of energy homeostasis and has been shown to influence mitochondrial biogenesis and oxidative phosphorylation [208]. ERRα on its own has been shown to associate with HIF transcriptional complexes and promote the glycolytic phenotype and angiogenesis in breast cancer [209].

It has also been demonstrated that forced depletion of ATP caused PGC-1α induction in cell culture via changes in intracellular Ca^2+^ [210]. Aberrant calcium flux was suggested as an inducer of PGC-1α expression, and changes in Ca^2+^ transients induced expression of VEGF [211]. Principal component analysis of transcription factor binding motifs in PGC-1α showed that the activator protein 1 complex (AP-1) is its crucial partner in eliciting the PGC-1α–controlled hypoxia response gene program [212]. Interestingly, elevated intracellular Ca^2+^ levels caused activation of AP-1 in hypoxia in a HIF-independent manner. Furthermore, it has been shown that reduced mitochondrial calcium uptake increased glutaminolysis and led to increased amounts of α-KG [213].

## 8. Conclusions

Our current understanding of how cancer cells can metabolically adapt to hypoxia has evolved a long way in the three decades since the discovery of HIF-1 in 1991 [214]. Despite the profound importance of this transcription factor in regulating multiple facets of hypoxic tumour metabolism, it is evident that in the absence of HIF-1, several adaptive mechanisms may compensate by remodelling metabolism at multiple levels of cellular organization in order to allow cancer cells not just to survive hypoxia, but also to proliferate, invade, and metastasise to distant organs. The available evidence so far has indicated that although cancer cells lacking HIF-1 have reduced expression of glycolytic enzymes, they still maintain their uptake of glucose via several mechanisms, including allosteric regulation, increased glucose transporter expression, and upregulation of creatine metabolism to maintain ATP levels.

Future work on potential anti-HIF resistance mechanisms should focus on examining the role of other forms of allosteric regulation, epigenetic reprogramming, post-translational modifications, spatial reorganization of glycolytic enzymes, and HIF-independent signalling pathways, such as the PI3K-Akt-mTOR, 2-HG, and AMPK pathways. Other metabolic pathways, such as glutaminolysis and lipogenesis, and transcription factors, such as Myc, may also be harnessed to enable the survival of such cells in periods of hypoxia. Given the currently available preclinical evidence, we postulate that cancer cell metabolic plasticity and redundancies in metabolic regulatory mechanisms mean that it is unlikely that targeting HIF-1 on its own will be sufficient to disrupt the metabolism of cancer cells and produce meaningful clinical benefit. As such, future work should also include looking for evidence of HIF-1 bypass in cancers treated with anti-HIF-1 drugs. The greatest benefit of agents targeting HIF-1 would probably be realized by using them in combination with other therapeutic agents that target these compensatory mechanisms.

## Data Availability

Not applicable.
